# Identification of long non-coding RNA in rice lines resistant to Rice blast pathogen Maganaporthe oryzae

**DOI:** 10.6026/97320630013249

**Published:** 2017-08-31

**Authors:** Priyanka Jain, Vinay Sharma, Himanshu Dubey, Pankaj Kumar Singh, Ritu Kapoor, Mandeeep Kumari, Jyoti Singh, Deepak V. Pawar, Deepak Bisht, Amolkumar U. Solanke, T.K. Mondal, T.R. Sharma

**Affiliations:** 1 ICAR-National Research Centre on Plant Biotechnology, Pusa Campus, New Delhi-110012, India;; 2 National Agri-Food Biotechnology Institute, Mohali, Punjab-140306, India; 3 Department of Bioscience & Biotechnology, Banasthali University, Tonk, Rajasthan-304022, India

**Keywords:** long non-coding RNA, rice lines resistant, blast pathogen, Maganaporthe oryzae

## Abstract

Rice blast disease caused by a fungus Magnaporthae oryzae is one of the most important biotic factors that severely damage the rice crop. Several molecular approaches are now being applied to tackle this issue in rice. It is of interest to study long non-coding RNA (lncRNA) in rice to control the disease. lncRNA, a non-coding transcript that does not encode protein, is known to play an important role in gene regulation of various biological processes. Here we describe a computational pipeline to identify lncRNA from a resistant rice line. The number of lncRNA found in resistant line was 1429, 1927 and 1981 in mock and M. oryzae (ZB13 and Zhong) inoculated samples, respectively. Functional classification of these lncRNA reveals a higher number of long intergenic non-coding RNA compared to antisense lncRNA in both mock and M. oryzae inoculated resistant rice lines. Many intergenic lncRNA candidates were identified from resistant rice line and their role to regulate the resistance mechanism in rice during M. oryzae invasion is implied.

## Background

Rice (Oryza sativa) is one of the important cereal crops cultivated and consumed throughout the world [1] and is a stable food for many countries, including India. In north India, severe losses in rice production occurs due to high pressure of blast disease because it affects both quantity as well quality of rice [2]. Though, many high yielding varieties of rice are available, the yield potential of these varieties is considerably affected by various biotic and abiotic stresses. Among the various biotic stresses like bacterial leaf blight, sheath blight and stem borer limiting the rice productivity, rice blast caused by Magnaporthe oryzae is a serious constraint in rice production at the global level. Blast pathogen infects the crop in all stages of its growth, starting from nursery to grain filling stage, under favourable environmental conditions. In depth analysis of transcriptome helps to understand mechanism of disease resistance that in turn help to decipher the disease etiology, progression and resistant breeding. Transcriptome studies in rice upon M. oryzae infection are few. The availability of very high quality genome sequences of both the rice and M. oryzae through the public genome databank makes an easy task to explore the transcriptome profiling using the RNA-seq data.

RNA transcribed from DNA does not have only to encode protein, but some RNAs that do not translated into proteins also have ability to regulate the gene expression. RNA molecules those are not encoded proteins are called as non-coding RNA (ncRNA). Nowadays, the involvement of these ncRNA in the regulation of gene expression has major attention of researchers to study them in various biotic and abiotic imposed conditions. The ncRNA are classified based on their size into small ncRNAs (sncRNAs) and long ncRNAs (lncRNAs). The sncRNAs are generally smaller than 200nt in length and further divided into microRNAs (mRNAs), small interfering RNAs (siRNAs), piwi-interacting RNAs, transacting siRNAs and natural antisense transcript siRNAs [3, 4].

However, lncRNAs are usually have more than 200 nt in length and divided into 3 groups according to position of nearby protein coding genes, i.e. long intergenic non-coding RNAs (lincRNAs), natural antisense transcripts (NATs) and intronic RNAs (incRNAs) [5]. In plants, the identification of lncRNA is more recent and not as comprehensive as compared to other eukaryotes [6,7] The ncRNAs have been reported to regulate the expression level of target genes via various molecular mechanisms [8, 9]. They also have a role in post-transcriptional modulations of mRNA processing. The lncRNAs are having little or no potential of encoding proteins, but they regulate the expression levels of target genes ranging from transcription to translation processes. Several evidences demonstrated that the plant lncRNAs have role in regulating the complex gene regulatory networks involved in plant development and stress management [7, 10, 11]. The genome-wide analysis of non-coding part of transcriptome uncovers lncRNA in maize and rice [12]. Integrating the genome wide association studies (GWAS) with the above finding showed hundreds of the long intergenic non-coding RNA (lincRNA) that contains SNPs related to genes of agriculturally important traits [12]. A few lncRNAs have been reported to regulate the developmental process in the plants, mainly during reproduction stage [13]. In rice two lincRNA genes, one LNC_Os03g44325 associated with seed color related-SNP and the other one LNC_Os05g27795 associated with leaf pubescence related-SNP have been identified [12]. In rice, there is no study performed till now to identify the lncRNA induced upon pathogen infection. The objective of this study was to identify the lncRNA induced upon M. oryzae infection in resistant rice lines.

## Methodology

### Dataset used for long -non coding RNA prediction

The RNA-seq data used for this study was downloaded from gene ontology omnibus (GEO) via an accession no. GSE62911. This accession GSE62911 contains 24 samples from resistant rice lines harbouring Pi9 blast resistance gene (Table 1). The detailed information related to these samples could be obtained through https://www.ncbi.nlm.nih.gov/geo/query/acc.cgi?acc=GSE62911 [14]. A total of 24 SRA files were downloaded for all samples of resistant rice lines. The SRA files were converted to fasta files using SRA toolkit [15]. The fasta files were applied for further analysis.

### Assembly and annotation of transcriptome data

**Figure 1:**
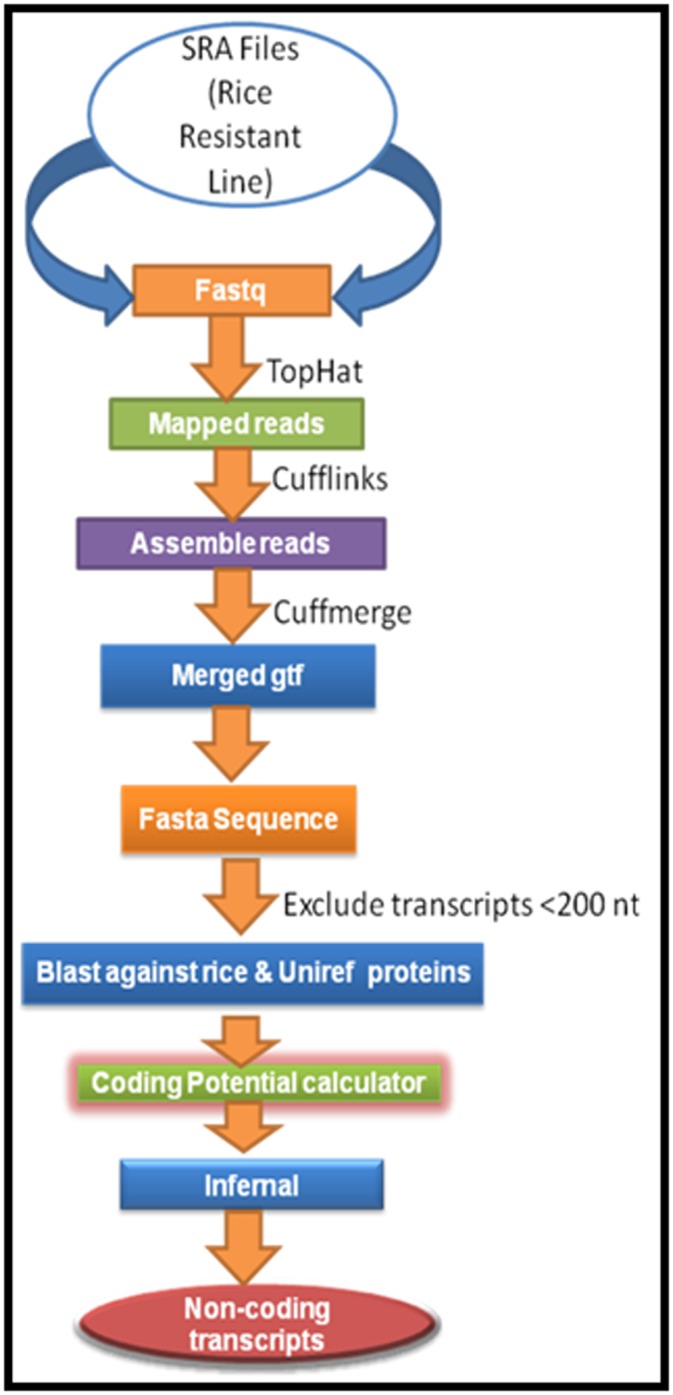
Computational pipeline to identify long non-coding RNA in rice.

A bioinformatics pipeline was developed in this study to extract long non-coding RNA from RNA-seq data of rice (Figure 1). All the 24 RNAseq reads were mapped to reference sequence of Oryza sativa japonica genome (MSU 7.0) using TopHat [16]. Cufflinks was employed to assemble the aligned reads [17]. Cuffmerge, a module of Cufflinks package was applied to merge all assembled (gtf) files obtained from Cufflinks. Then gffread function of Cufflinks was used to fetch fasta file from merged gtf file. Fasta files containing sequence of length less than 200 nucleotides were filtered. The filtered sequences were then blasted against known rice proteins. The hits of blast matches were considered by following different parameters, E-value ≤0.001, query coverage= 100 % and percent identity= 100%. The filtered sequences were also passed with CPC to find their coding potential [18]. The sequences reported to be non-coding by CPC were checked against Rfam database using cmscan in infernal [19] to detect any housekeeping RNAs (tRNA & rRNA) exist in the sequences.

### Analysis of long non-coding RNA

A perl script was used to fetch fasta sequence of transcript encoding as long non-coding RNA (lncRNA). The fasta and gtf files of each lncRNA were analyzed to determine the length of long noncoding RNA. The lncRNAs were categorized into different categories based on the position of protein coding genes, viz natural antisense lncRNA that overlap exon or intron in antisense orientation, long intergenic lncRNA and lncRNA overlapping gene in sense orientation using FEELnc [20]. The genomic location of each lncRNA identified from rice was represented using circos [21].

## Results and Discussion

In several species like Oryza sativa, Zea mays, Triticum aestivum, Actinidia chinensis, Gossypium arboreum and Brassica genomewide lncRNA has been identified and characterized [22, 23,24,25]. This is the first study in rice that reported the identification of lncRNA in resistant rice line with and without M. oryzae inoculation.

### Identification of lncRNAs in a blast resistant rice line

The pipeline developed to retrieve long coding RNA (lncRNA) was applied on 24 RNAseq data derived from a blast resistant rice line upon mock and M. oryzae (ZB13 and Zhong strain) inoculation. Three sets of each eight mock, eight ZB13 and eight Zhong replicates of resistant rice line from different time-points were mapped against the reference genome O. sativa japonica group, cultivar Nipponbare; MSU release 7 [26] with an average of above 93% of left and right aligned reads. Average concordant alignment rate of all the samples for paired end was 90 % (Table 2). All the assembled 24 gtf files were merged according to the data types, namely mock, ZB13 and Zhong using cuffmerge. After merging, three-merged gtf files, mock, ZB13 and Zhong were obtained. The fasta sequences of all transcripts present in these three gtf files were extracted. As we know that transcriptome data generated from total RNA captures all the transcripts that are present at transcriptional level.

So, the three fasta files had all the transcripts whether they have protein-coding potential or not. These three fasta files obtained from mock, ZB13 and Zhong were contained total 93326, 95671 and 94963 captured transcripts, respectively. After applying length (>200nt), blast (E-value ≤0.001, query coverage= 100 % and percent identity= 100%) and CPC filter criteria, total 1589, 2101 and 2038 transcripts were obtained from resistant line with mock, ZB13 and Zhong dataset, respectively. Furthermore, filter out the housekeeping RNA like tRNAs and rRNAs, the infernal (10-3 Evalue cutoff) and tRNA scan were applied to get the final numbers of long non-coding RNA. The final numbers of lncRNAs were 1429, 1927 and 1981 found in mock, ZB13 and Zhong dataset, respectively. These numbers clearly reflect that a higher lncRNAs were present in the datasets of resistant rice line challenged by M. oryzae strains compared to the dataset of mock inoculated. The identified lncRNA were further grouped into three categories, intergenic lncRNA, lnc overlapping exon or intron in sense orientation and lnc overlapping exon or intron in anti-sense orientation according to their relative locations from the nearest protein-coding genes. FEELnc classifier module classifies lncRNAs by employing a sliding window strategy. The lncRNAs with overlapping exon or intron in sense orientation were filtered from resistant line because major role of intergenic lncRNA and natural antisense lncRNA is known [22, 24, 27]. Total number of lncRNAs with overlapping exon in antisense orientation obtained from mock, ZB13 and Zhong datasets respectively were 218, 40 and 23.

**Figure 2:**
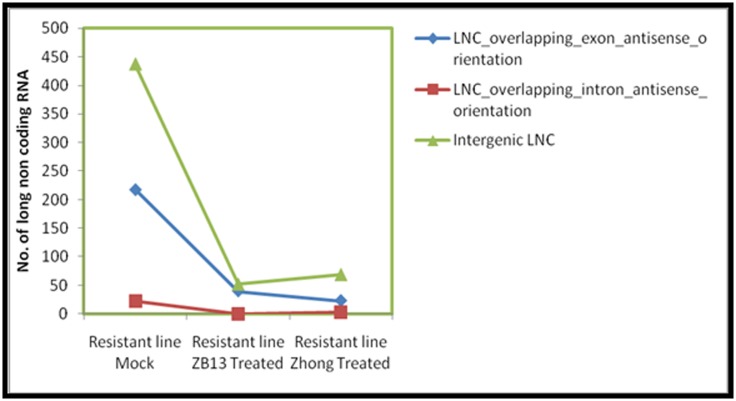
Number of long non-coding (lnc) RNA. Number of intergenic, overlapping exon or intron in antisense orientation lnc RNAs present in mock and M. oryzae (ZB13 and Zhong) inoculated resistant rice lines.

Similarly, total lncRNAs with overlapping intron in antisense orientation were also identified from these three datasets; mock, ZB13 and Zhong, i.e. 22, 0 and 3, respectively. Total numbers of intergenic lncRNAs (lincRNAs) were 437, 52 and 69 found in the mock, ZB13 and Zhong datasets, respectively (Figure 2). Among the classes of lncRNA, a maximum number of intergenic lncRNA was obtained in three datasets, followed by lncRNA with overlapping exon and intron in antisense orientations. The majority of lncRNAs identified in this study were belonged to lincRNAs (>64% of total lncRNAs) and it is consistent with the reports published by Zhang et al. (2014a) [28] and Li et al. (2014) [23], where a similar high proportion of intergenic lncRNAs in the total lncRNAs were found in rice (76%) and maize (93%), respectively. Several studies have been conducted to identify the candidate lncRNAs from the crop plants under various conditions. In order to study this, 3181 candidate lncRNAs responsive to Sclerotinia sclerotiorum infection were recognized from Brassica napus [25]. 664 transcripts were detected as drought-responsive lncRNAs from maize [29]. Many intergenic lncRNAs in response to phosphate starvation were also found in rice [22]. Zhang et al. (2014a) also discovered lncRNAs that show preferential expression during reproductive stages in rice. The lncRNAs identified in the resistant rice line upon M. oryzae treatment are important candidates that might play a pivotal regulatory role in the resistance mechanism of rice during biotic stress. Therefore, they have utility to be used for improving the crop yield.

### Characterization and genomic distribution of rice lncRNAs

**Figure 3:**
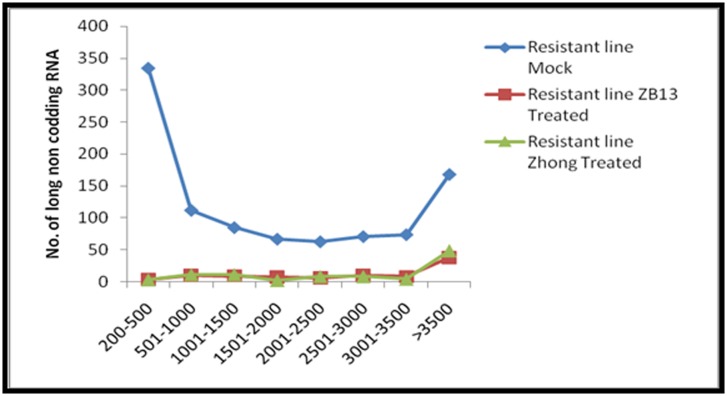
Length distribution of long non-coding RNA (lncRNA). Length variation of lncRNAs present in mock and M.oryzae (ZB13 and Zhong) inoculated resistant rice lines.

**Figure 4:**
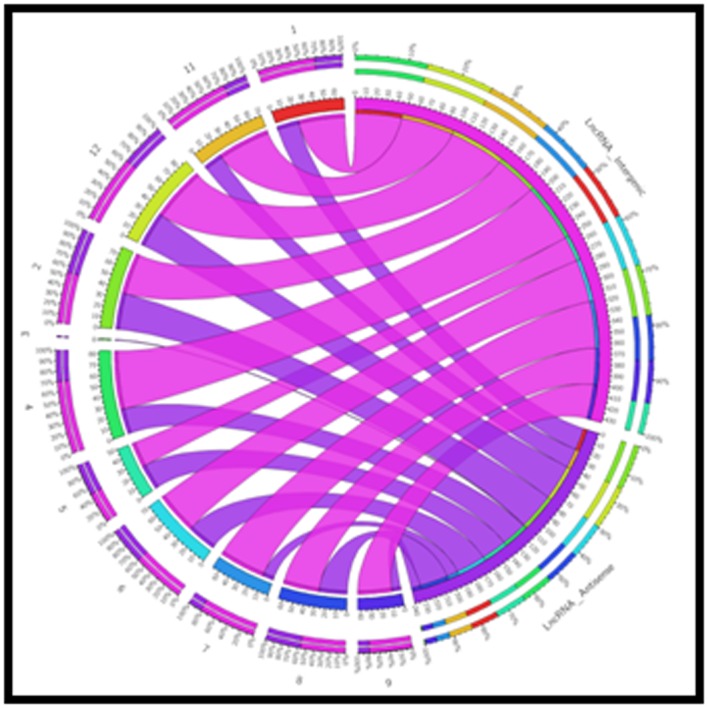
Distribution of lncRNAs along each rice chromosome in mock resistant line. The numbers of lncRNA intergenic are represented by outer pink ribbon and lncRNAs anti-sense is represented by inner blue ribbons.

The length distribution of intergenic lncRNA and antisense lncRNA show that highest number of lncRNAs were found in a range of 200 to 1000 nucleotides in all the three datasets, mock, ZB13 and Zhong (Figure 3). A recent study of Li et al. (2016) [30] also showed a high percentage of lncRNAs with length less than 1 kb. Genomic distribution of lncRNAs was performed to characterize them whether they are located in the form of a cluster or a particular chromosome has exceptionally high number of lncRNAs. The genomic distribution of lncRNA shows that both intergenic and antisense lncRNA were distributed across all the 12 chromosomes of rice, except 10th chromosome (Figure 4, 5 & 6). Similarly, Shuxia et al. (2017) also reported that lincRNA were evenly distributed across chromosomes of cassava plant. By determining the location of lncRNAs in the genome, its function can be predicted, since they mostly have function to regulate the nearest protein coding gene. There are several lncRNAs that are transcribed from within a protein coding gene locus and regulate their host genes functions. For example, an antisense lncRNA may inhibit the transcription of sense transcript of protein coding gene [31]. A related study was carried out in Gossypium arboretum by Zou et al. (2016) [24] to identify the lncRNAs that involved in fiber initiation and elongation processes. In this study, they found a total of 5,996 lncRNAs, of which 3,510 and 2,486 were classified as long intergenic non-coding RNAs (lincRNAs) and natural antisense transcripts (lncNAT), respectively. In another study conducted in Cassava, out of 682, 453 lncRNAs were lincRNAs transcribed under cold stress condition [27]. All the above findings clearly suggest that the lincRNAs have potential functional roles in plants to govern the gene expression level induced by various biotic and abiotic stresses.

## Conclusions

The computational pipeline developed in this study is relevant to find long non-coding RNAs (lncRNA) in different plants. The higher number of intergenic lncRNA found in resistant line compared to antisense lncRNA indicates the importance of intergenic lncRNA in resistant rice line upon biotic stress. The uniform distribution of lncRNA across all the rice chromosomes shows that there is no bias in chromosomal distribution of intergenic and antiense lncRNAs in mock and M. oryzae inoculated resistant line.

## Author’s Contribution

PJ, HD and PKS performed the complete analysis under guidance of TRS and VS. All authors contributed to the writing of the manuscript.

## Conflict of interests

The authors confirmed that this research article content has no conflict of interest.

## Figures and Tables

**Table 1:** Details of samples used for finding long non-coding RNA in resistant rice line (https://www.ncbi.nlm.nih.gov/geo/query/acc.cgi?acc=GSE62911)

Sample_name	Inoculation_type	Interaction	Phenotype
GSM1536133	inoculation without pathogen	no interaction	no change
GSM1536134	inoculation without pathogen	no interaction	no change
GSM1536135	ZB13 inoculation	Incompatible	Resistant
GSM1536136	ZB13 inoculation	Incompatible	Resistant
GSM1536137	Zhong inoculation	Incompatible	Resistant
GSM1536138	Zhong inoculation	Incompatible	Resistant
GSM1536145	inoculation without pathogen	no interaction	no change
GSM1536146	inoculation without pathogen	no interaction	no change
GSM1536147	ZB13 inoculation	Incompatible	Resistant
GSM1536148	ZB13 inoculation	Incompatible	Resistant
GSM1536149	Zhong inoculation	Incompatible	Resistant
GSM1536150	Zhong inoculation	Incompatible	Resistant
GSM1536157	inoculation without pathogen	no interaction	no change
GSM1536158	inoculation without pathogen	no interaction	no change
GSM1536159	ZB13 inoculation	Incompatible	Resistant
GSM1536160	ZB13 inoculation	Incompatible	Resistant
GSM1536161	Zhong inoculation	Incompatible	Resistant
GSM1536162	Zhong inoculation	Incompatible	Resistant
GSM1536169	inoculation without pathogen	no interaction	no change
GSM1536170	inoculation without pathogen	no interaction	no change
GSM1536171	ZB13 inoculation	Incompatible	Resistant
GSM1536172	ZB13 inoculation	Incompatible	Resistant
GSM1536173	Zhong inoculation	Incompatible	Resistant
GSM1536174	Zhong inoculation	Incompatible	Resistant

**Table 2:** Reads alignment and mapping on to the reference genome of resistant rice lines. R stands for replicate.

Sample Name	Left reads		Right reads		Aligned pairs	
	Input	Mapped	Input	Mapped	Multiple alignment	Concordant pair alignment
Resistant Line Mock R1	9680611	94.30%	9680611	94.40%	1.40%	91%
Resistant Line Mock R2	9617689	92.40%	9617689	94.30%	1.40%	90.70%
Resistant Line Mock R3	9057012	94.30%	9650070	94.50%	1.60%	91.20%
Resistant Line Mock R4	9602126	94.70%	9602126	94.40%	1.60%	91.10%
Resistant Line Mock R5	9636877	94.10%	9636877	94.30%	1.70%	90.40%
Resistant Line Mock R6	9636877	94.10%	9636877	94.30%	1.70%	90.40%
Resistant Line Mock R7	9591786	94.50%	9591786	93.50%	1.50%	89.10%
Resistant Line Mock R8	9712200	94.10%	9712200	94.30%	1.50%	90.40%
Resistant Line ZB13 Inoculated R1	9636099	94.10%	9636099	94.30%	1.40%	90.60%
Resistant Line ZB13 Inoculated R2	9652174	94.50%	9652174	94.50%	1.30%	91.00%
Resistant Line ZB13 Inoculated R3	9564527	92.20%	9564527	92.40%	1.60%	87.20%
Resistant Line ZB13 Inoculated R4	9565183	92.80%	9565183	92.60%	1.60%	88.00%
Resistant Line ZB13 Inoculated R5	9639040	93.90%	9639040	94.20%	2.00%	90.30%
Resistant Line ZB13 Inoculated R6	9717266	93.60%	9717266	93.80%	1.60%	89.90%
Resistant Line ZB13 Inoculated R7	9625362	94.10%	9625362	93.80%	1.80%	89.80%
Resistant Line ZB13 Inoculated R8	9620968	94.30%	9620968	94.60%	1.50%	90.90%
Resistant Line Zhong Inoculated R1	9645142	94.30%	9645142	94.30%	1.30%	91.00%
Resistant Line Zhong Inoculated R2	9627360	94.40%	9627360	94.50%	1.40%	90.90%
Resistant Line Zhong Inoculated R3	9612783	94.30%	9612783	94.00%	1.60%	90.30%
Resistant Line Zhong Inoculated R4	9602201	93.40%	9602201	93.50%	1.60%	89.30%
Resistant Line Zhong Inoculated R5	9613683	93.80%	9613683	93.80%	1.50%	89.80%
Resistant Line Zhong Inoculated R6	9555411	93.20%	9555411	93.30%	1.60%	89.20%
Resistant Line Zhong Inoculated R7	9670770	94.50%	9670770	94.40%	1.60%	91.10%
Resistant Line Zhong Inoculated R8	17226795	93.00%	17226795	92.60%	1.60%	88.20%

**Figure 5:**
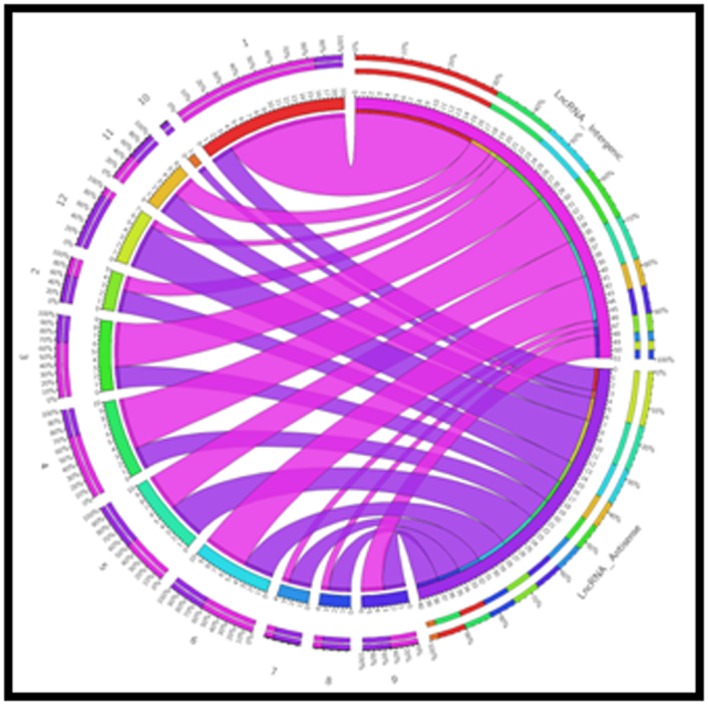
Distribution of lncRNAs along each rice chromosome in ZB13 inoculated resistant line. Outer pink ribbons represent the numbers of lncRNA intergenic and lncRNAs anti-sense is represented by inner blue ribbons.

**Figure 6:**
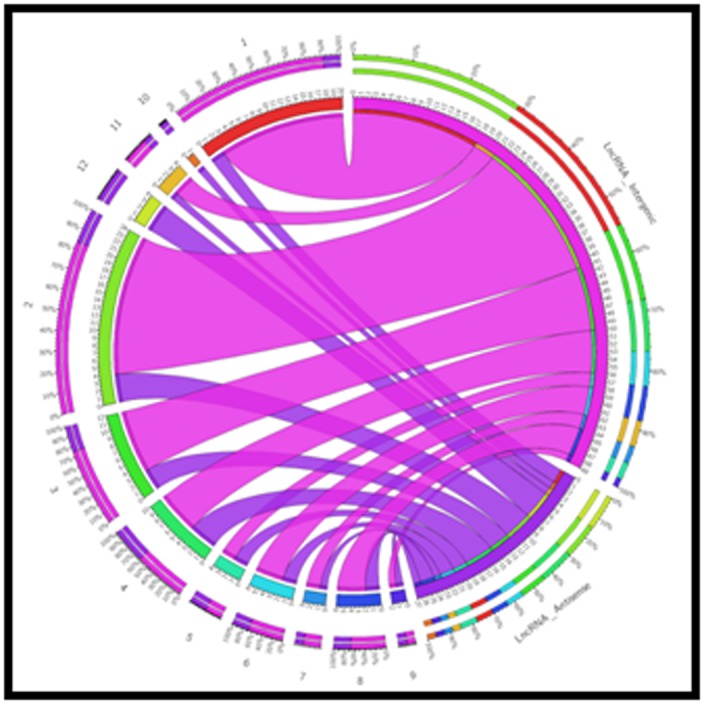
Distribution of lncRNAs along each rice chromosome in Zhong inoculated resistant line. Outer pink ribbons represent the numbers of lncRNA intergenic and lncRNAs anti-sense is represented by inner blue ribbons.
